# Invasive versus conservative strategy for older patients with myocardial infarction based on cognitive function: the SENIOR-RITA cognition analysis

**DOI:** 10.1093/ageing/afag330

**Published:** 2026-11-12

**Authors:** Kieran Gill, Robert F Storey, Colin Berry, David Newby, Keith A Fox, Stuart Pocock, Vijay Kunadian

**Affiliations:** Translational and Clinical Research Institute, Newcastle University, Newcastle upon Tyne, United Kingdom; Division of Clinical Medicine, NIHR Sheffield Biomedical Research Centre, Sheffield Teaching Hospitals NHS Foundation Trust, Sheffield, University of Sheffield, Sheffield, United Kingdom; University of Glasgow, Glasgow, United Kingdom; Centre for Cardiovascular Sciences, The University of Edinburgh Centre for Cardiovascular Science, Edinburgh, United Kingdom; University of Edinburgh, Edinburgh, United Kingdom; London School of Hygiene & Tropical Medicine, London, United Kingdom; Translational and Clinical Research Institute, Newcastle University, Newcastle upon Tyne, United Kingdom; Newcastle upon Tyne Hospitals NHS Foundation Trust- Cardiothoracic Centre, Freeman Hospital, Newcastle upon Tyne NE7 7DN, United Kingdom

**Keywords:** cognitive impairment, dementia, non-ST-segment elevation myocardial infarction, older adults, percutaneous coronary intervention

## Abstract

**Background:**

Older adults with non-ST-segment elevation myocardial infarction (NSTEMI) and cognitive impairment experience higher cardiovascular risk than patients with normal cognition. It remains unclear how cognition should influence whether older adults receive an invasive or conservative treatment strategy.

**Objectives:**

To compare an invasive and conservative treatment strategy among older adults with NSTEMI and cognitive impairment.

**Methods:**

This study is a pre-specified analysis of the SENIOR-RITA randomised controlled trial. Cognition was measured at randomisation using the Montreal Cognitive Assessment (MoCA). Patients were followed up for a median of 4.1 years postrandomisation. The primary outcome was a composite of time to cardiovascular death or nonfatal myocardial infarction (MI).

**Results:**

MoCA was available for 1455 patients (95.8%) who underwent randomisation. Within the cognitively impaired group (909 patients), the primary outcome occurred in 116 patients (26.8%) in the invasive group and in 141 patients (29.6%) in the conservative group (hazard ratio [HR], 0.85; 95% confidence interval [CI], 0.66 to 1.10; *P* = 0.20). Nonfatal MI occurred in 48 patients (11.1%) in the invasive group and in 75 patients (15.8%) in the conservative group (HR 0.64; 95% CI, 0.44 to 0.92). However, there was no interaction between treatment strategy and cognition.

**Conclusions:**

Among those with evidence of cognitive impairment at randomisation, an invasive treatment strategy did not reduce the primary composite outcome of cardiovascular death or nonfatal MI, and there was no evidence of treatment effect heterogeneity based on cognition. The reduction in nonfatal MI events among those with cognitive impairment should be interpreted as an exploratory finding.

## Key points

- Among older adults with NSTEMI, there was no evidence of treatment effect heterogeneity based on cognitive function.
- An invasive treatment strategy may reduce nonfatal MI among those with mild cognitive impairment.
- However, this possible reduction in nonfatal MI should be interpreted as an exploratory finding.
- There were few patients with moderate or severe cognitive impairment in our study, and shared-decision-making remains crucial.

## Introduction

Non-ST-segment elevation myocardial infarction (NSTEMI) accounts for 70% of myocardial infarction (MI) cases in older adults and affects around 20 000 older adults in the United Kingdom each year [1, 2]. Older adults frequently present with cognitive impairment, with a global prevalence of 23.7%, and even higher estimates among older adults hospitalised with MI [3, 4]. Cognitive impairment and cardiovascular disease are closely related, partly due to shared vascular risk factors, such as hypertension and diabetes [5, 6]. Cognitive impairment includes patients with dementia but also includes a significant proportion of patients who have mild cognitive impairment. Patients with mild cognitive impairment are at a three to five times higher risk of developing dementia than patients with normal cognition [7, 8]. Hence, strategies to reduce future cardiovascular events or hospitalisations in this population may be important to mitigate further cognitive or functional decline.

Observational studies highlight that older adults with NSTEMI and cognitive impairment are less likely to receive coronary angiography, and experience higher rates of major adverse cardiovascular events than those with normal cognition [4, 9–12]. However, it is not clear whether cognitive impairment at presentation should independently affect whether older adults receive an invasive or conservative treatment strategy, in the context of concomitant frailty and multimorbidity. This is because cognitive impairment has historically been a barrier in recruiting older patients to randomised controlled trial (RCTs) and contemporary RCTs in older adults with NSTEMI did not measure cognitive function at baseline [13–20].

This pre-specified analysis of the SENIOR-RITA trial focuses on cognitively impaired patients and aims to support decision making regarding an invasive or conservative treatment strategy for older adults with NSTEMI and cognitive impairment [21].

## Methods

The design and methods of the SENIOR-RITA trial have been published previously [21]. Briefly, the SENIOR-RITA trial was a prospective, multicentre, open-label, randomised controlled trial that recruited patients at least 75 years of age with NSTEMI to test an invasive versus a conservative treatment strategy. All patients provided informed consent. A consultee declaration was obtained from a family member or a caregiver for patients with cognitive impairment. This analysis was prespecified in the trial protocol and statistical analysis plan, which is available along with the full text of the SENIOR-RITA trial manuscript [21].

### Trial population

Patients with a clinical diagnosis of type 1 NSTEMI aged ≥75 years were enrolled. Exclusion criteria included diagnosis of ST-elevation MI, unstable angina, cardiogenic shock, or life expectancy of less than 1 year. Detailed inclusion and exclusion criteria are listed in the Supplemental Methods.

Montreal Cognitive Assessment (MoCA) was used to assess cognitive impairment at the time of randomisation [22]. Importantly, MoCA is a screening tool for cognitive impairment, rather than a diagnostic instrument for dementia. MoCA <26 indicates cognitive impairment and MoCA *≥*26 represents normal cognition, out of a maximum score of 30. MoCA assigns a differential weighting to seven different cognitive domains (executive function, naming, attention, language, abstraction, recall, orientation; Supplemental Methods) [22]. The standard education adjustment was applied where applicable (Supplemental Methods). MoCA <26 is designed to diagnose mild cognitive impairment with a sensitivity and specificity of 90% and 87% respectively [23]. MoCA 18–25 indicates mild cognitive impairment, MoCA 10–17 indicates moderate cognitive impairment and MoCA <10 represents severe cognitive impairment. Frailty was measured using the Fried Frailty Index and Rockwood Frailty Index [24]. Charlson Comorbidity Index (CCI) was used to quantitatively assess multimorbidity [25].

### Randomisation and treatment

All patients were randomly assigned in a 1:1 ratio to an invasive treatment with revascularisation if appropriate, plus optimal medical therapy (the invasive-strategy group), or to a conservative management with optimal medical therapy alone (conservative-strategy group). Randomisation was on 1:1 basis using a variable-length block stratified method with randomly selected block sizes of two, four, six and eight. Randomisation was performed at each site using a secure web-based system.

Optimal medical therapy included aspirin (75 mg daily), a P2Y12 receptor antagonist, a beta-blocker (target heart rate of 60 to 70 beats per minute), statin therapy, and an angiotensin-converting enzyme inhibitor or angiotensin II receptor blocker. Comorbidities such as hypertension, diabetes and hypercholesterolaemia were managed according to the clinical practice guidelines.

Patients assigned to the invasive group underwent coronary angiography with coronary revascularisation if appropriate and received optimal medical therapy. Coronary angiography was conducted following local guidelines. Coronary revascularisation was carried out within 3 to 7 days, when appropriate, through either percutaneous coronary intervention or coronary artery bypass grafting, as determined by the attending cardiologist and the multidisciplinary team. In the conservative strategy group, coronary angiography was permitted in cases of clinical deterioration when deemed necessary by the treating physicians.

### Outcomes and follow up

The primary composite outcome was time to cardiovascular death or nonfatal MI defined by the fourth universal definition of MI [26]. Secondary outcome measures were a composite of all-cause death or MI, all-cause death, cardiovascular death, noncardiovascular death, recurrent MI, subsequent coronary angiography, subsequent coronary revascularisation, hospitalisation for heart failure, stroke, transient ischaemic attack and bleeding (as per the Bleeding Academic Research Consortium Criteria) [27]. Safety outcomes included procedural and in-hospital complications occurring in patients assigned to the invasive strategy. For this pre-specified subgroup analysis, we performed stratified analyses on primary and secondary outcomes based on MoCA score. Site level follow-up was performed at 6 months, 1 year and then yearly until 5 years either via telephone or in person visit.

### Statistical analysis

Baseline characteristics were compared between treatment strategies and between cognitive impairment versus normal cognition groups using the Chi-squared test, Fisher’s exact test or Mann–Whitney U test as required. Prespecified clinical outcomes were analysed in accordance with the statistical analysis plan of SENIOR-RITA [21]. Our analysis was performed according to the intention-to-treat principle.

Our pre-specified subgroup analysis focused on a dichotomous comparison between MoCA <26 and MoCA *≥*26 as per the main SENIOR-RITA publication and protocol [21]. Cumulative incidence was estimated from the Kaplan–Meier time-to-event function. For patients who experienced both components of the primary composite outcome, time-to-first event was used. Patients who did not experience the event of interest and patients with missing primary outcome codes (<0.5%) were censored. Treatment strategies were compared using the log-rank test. Treatment effect was calculated using the Cox proportional hazards model to obtain hazard ratios with 95% confidence intervals, adjusted for Rockwood Frailty Scale and CCI at randomisation. The proportional hazards assumption was tested by plotting the Schoenfeld residuals against time. For outcomes where the proportional hazards assumption was violated, the difference in the restricted mean event-free time was calculated at 5 years post-randomisation. Sensitivity analyses were performed for the primary outcome to account for competing risk (using a Fine and Grey subdistribution hazard model) and missing MoCA data (using multiple imputation chained equations). Interaction testing was conducted between treatment strategy and cognitive function for the primary outcome and its components to test for subgroup differences.

Treatment interactions with cognition as a continuous variable were analysed using restricted cubic splines for the primary outcome and its components, adjusted for Rockwood Clinical Frailty Scale at baseline. We also stratified analyses according to normal cognition, mild cognitive impairment and moderate or severe cognitive impairment. These analyses were conducted post-hoc and should be interpreted as exploratory findings.

All analyses were conducted using R version 4.5.1 and R Studio version 2025.05.1, using the packages ‘survival’, ‘mice’, ‘ggplot2’, ‘survminer’, ‘survRM2’, ‘rms’ and ‘interact-ionRCS’. For all analyses, a two-tailed P-value <0.05 was set a priori as the threshold for statistical significance. No adjustments for multiplicity were made.

## Results

### Patients

In total, 6977 eligible participants were screened, of whom 1518 underwent randomisation. Patients were recruited from 48 National Health Service (NHS) hospitals in England and Scotland between November 2016 and March 2023. Cognition was formally assessed at randomisation using MoCA, which was available for 1455 of the 1518 randomised patients (95.8%), Figure S1. Baseline characteristics of patients stratified by cognitive function are shown in Tables 1 and S1.

**Table 1:** Baseline demographics and clinical characteristics of cognitively impaired and normal cognition patients.

Characteristics	Cognitive impairment (*N* = 909)	Normal cognition (*N* = 546)	*P* value
Female sex—no. (%)	426 (47)	227 (42)	0.050
Median Age—yr (IQR)	83 (79–86)	81 (78–84)	<0.001
≥75 to <80—no. (%)	242 (27)	196 (36)	
≥80 to <85—no. (%)	352 (39)	217 (40)	
≥85 to <90—no. (%)	236 (26)	107 (20)	
≥90 to <95—no. (%)	72 (7.9)	20 (3.7)	
≥95—no. (%)	7 (0.8)	6 (1.1)	
Median days from admission to randomisation (IQR)	2 (1–3)	2 (1–3)	0.041
Median Rockwood Frailty score (IQR)	3 (2–5)	3 (2–4)	<0.001
Frail (Rockwood score ≥ 5)—no./total no. (%)	239/909 (26.3)	55/546 (10.1)	
Very fit (1) - no./total no. (%)	84/909 (9.2)	113/546 (21)	
Well, without active disease (2) - no./total no. (%)	149/909 (16)	126/546 (23)	
Well, with treated co-morbidities (3) - no./total no. (%)	236/909 (26)	166/546 (30)	
Apparently vulnerable (4) - no./total no. (%)	201/909 (22)	86/546 (16)	
Mildly frail (5) - no./total no. (%)	155/909 (17)	40/546 (7.3)	
Moderately frail (6) - no./total no. (%)	74/909 (8.1)	12/546 (2.2)	
Severely frail (7) - no./total no. (%)	10/909 (1.1)	3/546 (0.5)	
Median Fried Frailty score (IQR)	2 (1–3)	1 (0–2)	<0.001
Robust (0) - no./total no. (%)	145/875 (17)	155/533 (29)	
Pre-frail (1 or 2) - no./total no. (%)	405/875 (46)	256/533 (48)	
Fried Frailty score Frail (≥3) - no./total no. (%)	325/875 (37)	122/533 (23)	
Median Charlson age-adjusted co-morbidity index (IQR)	5 (5–7)	5 (4–6)	<0.001
Smoking status			0.3
Current smoker—no./total no. (%)	45/901 (5.0)	30/542 (5.5)	
Ex-smoker—no./total no. (%)	403/901 (45)	262/542 (48)	
Never smoked—no./total no. (%)	453/901 (50)	250/542 (46)	
Median GRACE 2 Score (IQR)	136 (124–150)	133 (121–148)	0.005
Hypertension—no./total no. (%)	606/909 (67)	347/546 (64)	0.2
Diabetes—no./total no. (%)	269/909 (30)	173/546 (32)	0.4
Hypercholesterolaemia—no./total no. (%)	276/908 (30)	183/545 (34)	0.2
History of chronic kidney disease—no./total no. (%)	197/909 (22)	97/546 (18)	0.072
Previous myocardial infarction—no./total no. (%)	312/909 (34)	144/546 (26)	0.002
Previous PCI—no./total no. (%)	199/909 (22)	90/545 (17)	0.013
Previous CABG—no./total no. (%)	110/909 (12)	63/546 (12)	0.7
History of peripheral vascular disease—no./total no. (%)	70/909 (7.7)	38/546 (7.0)	0.6
History of TIA/stroke—no./total no. (%)	155/909 (17)	65/546 (12)	0.008
History of COPD—no./total no. (%)	149/909 (16)	69/546 (13)	0.052
History of congestive heart failure—no./total no. (%)	104/909 (11)	32/546 (5.9)	<0.001
History of dementia—no./total no. (%)	28/909 (3.1)	1/546 (0.2)	<0.001

IQR denotes interquartile range, MoCA denotes Montreal Cognitive Assessment, PCI denotes percutaneous coronary intervention, CABG denotes coronary artery bypass graft, TIA denotes transient ischaemic attack and COPD denotes chronic obstructive pulmonary disease.

The cognitively impaired group included 909 patients, of whom 426 patients (47%) were female. Within the cognitively impaired group, the majority 754/909 (83%) had mild cognitive impairment, 132/909 (14.5%) had moderate cognitive impairment and 23/909 (2.5%) had severe cognitive impairment, Table S1, Figure S2. Cognitively impaired patients were, on average, older than those with normal cognition (83 years [IQR 79–86] vs. 81 years [IQR 78–84]). In addition, a greater percentage of cognitively impaired patients were frail than those with normal cognition according to both Rockwood and Fried Frailty scores (Rockwood score *≥*5: 239/909 [26.3%] vs. 55/546 [10.1%]; Fried Frailty score *≥*3: 325/875 [37%] vs. 122/533 [23%]).

Baseline characteristics, medical therapy and procedural characteristics as per the randomised strategy among cognitively impaired and normal cognition patients respectively are shown in Tables 2 and S2-S4.

**Table 2:** Baseline demographics and clinical characteristics of cognitively impaired patients as per randomised strategy.

Characteristics	Invasive strategy (*N* = 433)	Conservative strategy (*N* = 476)	*P* value
Female sex—no. (%)	204 (47)	222 (47)	0.9
Median Age – yr (IQR)	83 (80–86)	82 (79–86)	0.2
≥75 to <80—no. (%)	103 (24)	139 (29)	0.4
≥80 to <85—no. (%)	176 (41)	176 (37)	
≥85 to <90—no. (%)	115 (27)	121 (25)	
≥90 to <95—no. (%)	35 (8.1)	37 (7.8)	
≥95—no. (%)	4 (0.9)	3 (0.6)	
Median days from admission to randomisation (IQR)	2 (1–3)	2 (1–3)	0.2
Median Rockwood Frailty score (IQR)	4 (2–5)	3 (2–5)	0.9
Frail (Rockwood score ≥ 5)—no./total no. (%)	110/433 (25.4)	129/476 (27.1)	
Very fit (1)—no./total no. (%)	41/433 (9.5)	43/476 (9.0)	
Well, without active disease (2)—no./total no. (%)	70/433 (16)	79/476 (17)	
Well, with treated co-morbidities (3) —no./total no. (%)	105/433 (24)	131/476 (28)	
Apparently vulnerable (4) - no./total no. (%)	107/433 (25)	94/476 (20)	
Mildly frail (5)—no./total no. (%)	72/433 (17)	83/476 (17)	
Moderately frail (6)—no./total no. (%)	34/433 (7.9)	40/476 (8.4)	
Severely frail (7)—no./total no. (%)	4/433 (0.9)	6/476 (1.3)	
Median Fried Frailty score (IQR)	2 (1–3)	2 (1–3)	0.9
Robust (0)—no./total no. (%)	70/412 (17)	75/463 (16)	0.7
Pre-frail (1 or 2)—no./total no. (%)	188/412 (46)	217/463 (47)	
Fried Frailty score Frail (≥3) — no./total no. (%)	154/412 (37)	171/463 (37)	
Median Charlson age-adjusted co-morbidity index (IQR)	5 (5–7)	5 (4–7)	0.4
Smoking status			0.023
Current smoker—no./total no. (%)	14/430 (3.3)	31/471 (6.6)	
Ex-smoker—no./total no. (%)	207/430 (48)	196/471 (42)	
Never smoked—no./total no. (%)	209/430 (49)	244/471 (52)	
Median GRACE 2 Score (IQR)	137 (125–150)	136 (123–150)	0.7
Hypertension—no./total no. (%)	287/433 (66)	319/476 (67)	0.8
Diabetes—no./total no. (%)	124/433 (29)	145/476 (30)	0.5
Hypercholesterolaemia—no./total no. (%)	141/433 (33)	135/475 (28)	0.2
History of chronic kidney disease—no./total no. (%)	96/433 (22)	101/476 (21)	0.7
Previous myocardial infarction—no./total no. (%)	162/433 (37)	150/476 (32)	0.061
Previous PCI—no./total no. (%)	104/433 (24)	95/476 (20)	0.14
Previous CABG—no./total no. (%)	64/433 (15)	46/476 (9.7)	0.018
History of peripheral vascular disease—no./total no. (%)	34/433 (7.9)	36/476 (7.6)	0.9
History of TIA/stroke—no./total no. (%)	86/433 (20)	69/476 (14)	0.032
History of COPD—no./total no. (%)	71/433 (16)	78/476 (16)	>0.9
History of congestive heart failure—no./total no. (%)	56/433 (13)	48/476 (10)	0.2
History of dementia—no./total no. (%)	16/433 (3.7)	12/476 (2.5)	0.3

IQR denotes interquartile range, MoCA denotes Montreal Cognitive Assessment, PCI denotes percutaneous coronary intervention, CABG denotes coronary artery bypass graft, TIA denotes transient ischaemic attack and COPD denotes chronic obstructive pulmonary disease

### Primary outcome and its components

Among cognitively impaired patients, the primary outcome occurred in 116 patients (26.8%) in the invasive group and in 141 patients (29.6%) in the conservative group (hazard ratio [HR], 0.85; 95% confidence interval [CI], 0.66 to 1.10; *P* = 0.20; Table 3 and Figure 1). Among those with normal cognition, the primary outcome occurred in 65 patients (22.3%) in the invasive group and in 48 patients (18.8%) in the conservative group (HR, 1.26; 95% CI, 0.86 to 1.84; Table 3 and Figure S3). With respect to the primary outcome, the proportional hazards assumption of the Cox model was violated. Restricted mean event-free time analysis for nonproportional hazards among patients with cognitive impairment and normal cognition are shown in Table S6-S7. The Cox proportional hazards model for the primary outcome remained consistent when performing sensitivity analyses for competing risk and missing MoCA data (Table S8).

**Table 3:** Primary and secondary outcomes.

Outcome variables in cognitively impaired patients	Invasive strategy^a^ (*N* = 433)	Conservative strategy^*^ (*N* = 476)	Treatment effect hazard ratio^b^ 95% CI	*P* value^c^
Primary outcome and its components: Cardiovascular death or nonfatal MI^b^—no. (%)	116 (26.8)	141 (29.6)	0.85 (0.66–1.10)	0.20
Cardiovascular death—no. (%)	80 (18.5)	82 (17.2)	1.02 (0.75–1.40)	0.89
Nonfatal MI^b^—no. (%)	48 (11.1)	75 (15.8)	0.64 (0.44–0.92)	0.017
Secondary outcomes				
All cause death or nonfatal MI—no. (%)	196 (45.3)	224 (47.1)	0.89 (0.73–1.08)	0.25
All cause death^b^—no. (%)	173 (40.0)	180 (37.8)	1.01 (0.82–1.25)	0.91
Noncardiovascular death^b^—no. (%)	93 (21.5)	97 (20.4)	1.00 (0.75–1.34)	0.99
Fatal or nonfatal MI^b^—no. (%)	56 (12.9)	83 (17.4)	0.68 (0.48–0.96)	0.028
Subsequent coronary angiography—no. (%)	19 (4.4)	102 (21.4)	0.18 (0.11–0.29)	<0.01
Subsequent coronary revascularisation^b^—no. (%)	12 (2.8)	57 (12.0)	0.22 (0.12–0.41)	<0.01
Stroke^b^—no. (%)	17 (3.9)	29 (6.1)	0.56 (0.30–1.04)	0.068
Transient ischaemic attack^b^—no. (%)	10 (2.3)	7 (1.5)	1.69 (0.64–4.49)	0.29
Hospitalisation for heart failure^b^—no. (%)	53 (12.2)	51 (10.7)	1.12 (0.76–1.65)	0.55
Bleeding (BARC Type 2+) ^†^ − no. (%)	34 (7.9)	30 (6.3)	1.24 (0.76–2.03)	0.40
Outcome variables in normal cognition patients	Invasive strategy^a^ (*N* = 291)	Conservative strategy^a^ (*N* = 255)	Treatment effect hazard ratio^b^ 95% CI	*P* value^c^
Primary outcome and its components: Cardiovascular death or nonfatal MI^b^—no. (%)	65 (22.3)	48 (18.8)	1.26 (0.86–1.84)	0.24
Cardiovascular death^b^—no. (%)	31 (10.7)	20 (7.8)	1.34 (0.76–2.35)	0.31
Nonfatal MI^b^—no. (%)	36 (12.4)	33 (12.9)	0.98 (0.61–1.59)	0.94
Secondary outcomes				
All cause death or nonfatal MI^b^—no. (%)	105 (36.1)	81 (31.8)	1.15 (0.85–1.55)	0.36
All cause death^b^—no. (%)	83 (28.5)	54 (21.2)	1.38 (0.96–1.99)	0.078
Noncardiovascular death^b^—no. (%)	52 (17.9)	34 (13.3)	1.23 (0.79–1.91)	0.36
Fatal or nonfatal MI^b^—no. (%)	40 (13.7)	34 (13.3)	1.00 (0.64–1.64)	0.91
Subsequent coronary angiography—no. (%)	20 (6.9)	72 (28.2)	0.21 (0.13–0.34)	<0.01
Subsequent coronary revascularisation^b^—no. (%)	13 (4.5)	39 (15.3)	0.28 (0.15–0.53)	<0.01
Stroke^b^—no. (%)	10 (3.4)	9 (3.5)	1.01 (0.40–2.56)	0.99
Transient ischaemic attack^b^—no. (%)	7 (2.4)	2 (0.78)	2.89 (0.59–14.10)	0.19
Hospitalisation for heart failure^c^—no. (%)	25 (8.6)	26 (10.2)	0.83 (0.47–1.48)	0.53
Bleeding (BARC Type 2+)^c^ —no. (%)	21 (7.2)	17 (6.7)	1.04 (0.55–1.99)	0.90

BARC denotes Bleeding Academic Research Consortium, MI denotes Myocardial infarction and CI denotes Confidence Interval.

Hazard ratios are derived from Cox proportional hazard models, adjusted for Rockwood Frailty Scale and Charlson Comorbidity Index at randomisation

^a^Data shown are the number of patients with at least one event (percent)

^b^Analyses satisfied the proportional hazards assumption of the Cox model.

^c^ *P* values were calculated using a log-rank test

**Figure 1:**
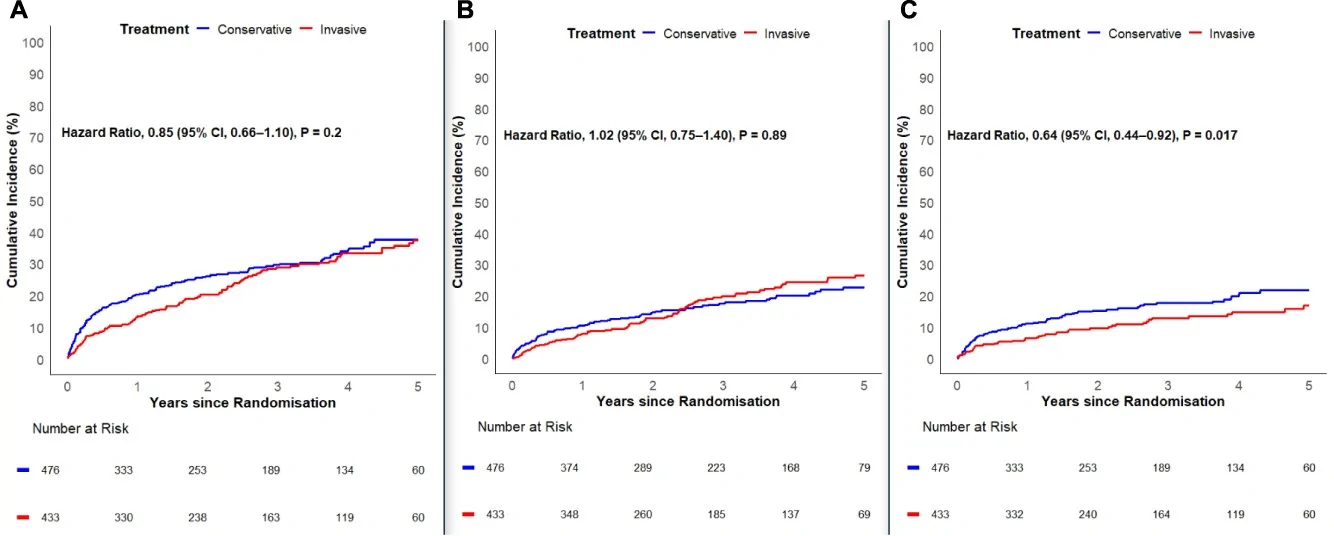
Cumulative incidence of the primary outcome and its components among cognitively impaired patients. Panel A: Cumulative incidence of the primary composite outcome of cardiovascular death or nonfatal myocardial infarction among cognitively impaired patients. Panel B: Cumulative incidence of cardiovascular death among cognitively impaired patients. Panel C: Cumulative incidence of nonfatal myocardial infarction among cognitively impaired patients. Abbreviations: CI: confidence interval.

Among patients with evidence of cognitive impairment at randomisation, cardiovascular death occurred in 80 patients (18.5%) in the invasive group and in 82 patients (17.2%) in the conservative group (HR, 1.02; 95% CI, 0.75 to 1.40; Table 3 and Figure 1). Among those with normal cognition, cardiovascular death occurred in 31 patients (10.7%) in the invasive group and in 20 patients (7.8%) in the conservative group (HR, 1.34; 95% CI, 0.76 to 2.35; Table 3 and Figure S4).

Among patients with cognitive impairment, nonfatal MI occurred in 48 patients (11.1%) in the invasive group and in 75 patients (15.8%) in the conservative group (HR, 0.64; 95% CI, 0.44 to 0.92; Table 3 and Figure 1). Among patients with normal cognition, nonfatal MI occurred in 36 patients (12.4%) in the invasive group compared to 33 patients (12.9%) in the conservative group (HR, 0.98; 95% CI, 0.61 to 1.59; Table 3 and Figure S5). Despite these numerical differences, there was no interaction between treatment strategy and cognitive function with respect to the primary outcome and its components (Figure S6).

When cognition was measured as a continuous variable, there were no interactions between treatment strategy and cognition with respect to the primary outcome and its components, after adjustment for Rockwood Clinical Frailty Scale at baseline. The restricted cubic spline for the primary composite outcome shows that treatment effect was relatively consistent across MoCA scores, providing further evidence of no treatment effect heterogeneity based on cognition (Figure 2). The restricted cubic spline for nonfatal MI suggests that the numerical reduction in nonfatal MI events was predominantly for patients with mild cognitive impairment and attenuated at MoCA scores <20 (Figure 2). Hazard ratios stratified by mild cognitive impairment and moderate or severe cognitive impairment showed broadly consistent results. There were no signals of increased harm among patients with moderate or severe cognitive impairment undergoing an invasive treatment strategy (Table S5).

**Figure 2:**
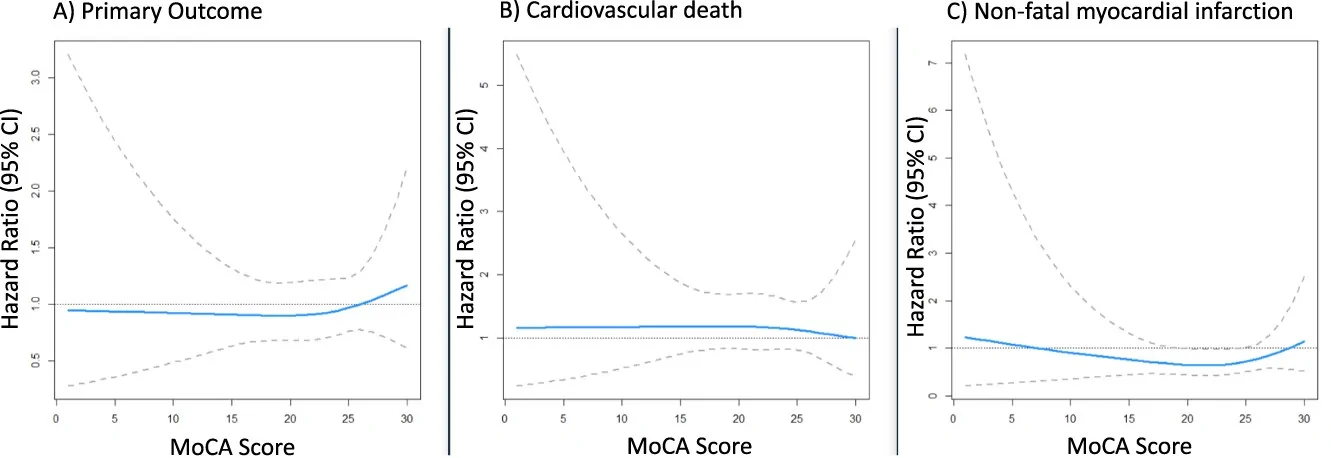
Restricted cubic splines to analyse the interaction between treatment strategy and cognition with respect to the primary outcome and its components. Panel A: Restricted cubic spline to show the interaction between treatment strategy and cognition with respect to the primary composite outcome of cardiovascular death of nonfatal myocardial infarction. Panel B: Restricted cubic spline to show the interaction between treatment strategy and cognition with respect to cardiovascular death. Panel C: Restricted cubic spline to show the interaction between treatment strategy and cognition with respect to nonfatal myocardial infarction. ^†^Adjusted for Rockwood Clinical Frailty Scale at baseline. Abbreviations: CI: confidence interval; MoCA: Montreal Cognitive Assessment.

### Secondary outcomes

No differences were found in most secondary outcomes between invasive and conservative groups among patients with cognitive impairment and normal cognition, except for a possible reduction in fatal or nonfatal MI among patients with cognitive impairment (HR, 0.70; 95% CI, 0.50 to 0.98; Table 3). Subsequent coronary angiography (HR, 0.18; 95% CI, 0.11 to 0.29 and HR, 0.21; 95% CI, 0.13 to 0.34; Table 3) and subsequent coronary revascularisation (HR, 0.22; 95% CI, 0.12 to 0.41 and HR, 0.28; 95% CI, 0.15 to 0.53; Table 3) occurred less in both cognitively impaired and normal cognition patients who received an invasive treatment strategy.

### Procedural complications

Overall, there were no significant differences in any recorded procedural complications between cognitively impaired and normal cognition patients (Table S9). Bleeding complications were 1.4% for both cognitively impaired and normal cognition patients undergoing coronary angiography. The incidence of other complications such as coronary dissection, increase in serum creatinine concentration *≥*25%, vascular complications requiring intervention and confusion or agitation were each <1% for cognitively impaired and normal cognition groups.

## Discussion

This pre-specified secondary analysis of the SENIOR-RITA trial compared an invasive versus conservative treatment strategy, stratified by MoCA score, over a median follow-up of 4.1 years. Among older patients with NSTEMI and cognitive impairment, an invasive treatment strategy did not reduce the primary composite outcome of cardiovascular death or nonfatal MI. There was no interaction between treatment strategy and cognition.

In SENIOR-RITA, the cognitive impairment subgroup accounted for 62.5% of the total population, most of whom had mild cognitive impairment (83%). A minority (3%) had dementia or severe cognitive impairment at baseline. Previous observational studies, such as ICON-1 and SILVER-AMI, estimate the prevalence of cognitive impairment among hospitalised MI patients to be 48% and 17%, respectively [4, 9]. Our higher prevalence of cognitive impairment may be related to the high sensitivity of MoCA for mild cognitive impairment. Transient stressors or delirium may have temporarily depressed MoCA scores at randomisation. For example, a prospective registry study estimated that 7% of older patients with NSTEMI present with delirium, although we proactively excluded patients with predisposing factors to delirium (such as acute infection) [21, 28]. Patients with mild cognitive impairment still represent a clinically important population who are at higher risks of cardiovascular death and all-cause mortality than patients with normal cognition [4, 9, 10].

Both SENIOR-RITA and an individual patient data meta-analysis consisting of six, small RCTs incorporating 1479 older patients with NSTEMI showed that an invasive treatment strategy resulted in no difference in cardiovascular death, but a reduction in nonfatal MI, compared to a conservative treatment strategy [21, 29]. Whether these results can be applied to cognitively impaired patients has been unclear, as cognition has not been formally assessed in any previous RCT [29, 30]. Our analysis suggests that these findings can be applied to patients with mild cognitive impairment, but not necessarily those with more advanced cognitive impairment. Our primary outcome result remained consistent when accounting for noncardiovascular death as a competing risk. This strengthens the generalisability of our findings to a realistic geriatric population where frailty, multimorbidity and cognitive impairment coexist [2, 31–34].

Holistic goals of care that consider quality of life, functional independence and days alive and out of hospital are crucial to older adults [11]. Among patients with evidence of cognitive impairment at randomisation, an invasive treatment strategy was associated with a 36% reduction in nonfatal MI events compared to a conservative treatment strategy. Restricted cubic spline analysis suggests this benefit was observed in patients with mild cognitive impairment and attenuated at lower cognitive scores (indicating more severe cognitive impairment). However, given that there was no formal interaction between treatment strategy and cognition, this finding should be interpreted as exploratory. Subsequent MI may increase the risk of cognitive decline post-NSTEMI [4, 35]. Preventing further cognitive decline is crucial for older adults with mild cognitive impairment, due to their markedly higher, but potentially reversible risk of dementia [7, 8]. Our data does not provide evidence of a reduction in nonfatal MI events among those with moderate or severe cognitive impairment, however, these patients account for a small proportion of the cognitively impaired subgroup. Hence, our analysis is likely underpowered to assess treatment effect heterogeneity among those with more severe cognitive impairment. Furthermore, it is possible that patients with severe cognitive impairment may have been excluded due to clinical decision.

Notably, an invasive treatment strategy among cognitively impaired patients did not increase the risk of BARC 2+ bleeding when compared to a conservative treatment strategy, which is a common concern among older adults. Combined with the low overall percentage of procedural complications, this emphasises that older adults with NSTEMI and cognitive impairment should not be disadvantaged from an invasive treatment strategy due to perceived harm [36]. A shared decision-making approach should be adopted, individualised to the relevant goals of care for each older adult and their caregivers.

Our results among patients with normal cognition show that an invasive treatment strategy was associated with a numerically higher cumulative incidence of the primary outcome. Angiographic and procedural characteristics, including multivessel disease, were similar between cognitively impaired and normal cognition subgroups. Secondary prevention medications on discharge were also similar between cognitively impaired and normal cognition groups, although 51% of patients with normal cognition were discharged on a potent P2Y_12_ receptor antagonist, compared to 38% in the cognitively impaired group.

Key strengths are that this secondary analysis was prespecified, and SENIOR-RITA is the first randomised controlled trial in this context to routinely measure cognitive function. Crucially, our study measured cognitive function at a critical time point when treatment decisions are being made, and we were able to assess baseline cognition in 95.8% of patients at randomisation. This also reinforces the feasibility of routine administration of MoCA in an acute NSTEMI setting. Moreover, the cognitively impaired subgroup consisted of a representative proportion of female patients, frail patients and patients with comorbidities, capturing the association between these baseline characteristics in clinical practice [6, 37–39]. Many of these patients have been previously underrepresented in clinical trials [40]. Additionally, SENIOR-RITA is the largest randomised controlled trial in older adults with NSTEMI, providing relevant data on long-term outcomes for older adults and their families or caregivers, to guide shared decision-making [21].

Limitations include the distribution of MoCA scores clustered between 20 and 30. Our analysis mostly represents patients with mild cognitive impairment, which limits the interpretation of treatment effects for patients with moderate or severe cognitive impairment. In SENIOR-RITA, the screening to randomisation ratio was 1518/6977 (22%). Cognition was not measured for patients who were screened but not randomised, therefore it is unclear whether patients with severe cognitive impairment were excluded from SENIOR-RITA due to clinical decision [41]. However, the mean age, percentage of frail patients and percentage of female patients was similar between the two groups [2]. Furthermore, the median time from admission to angiography in the cognitively impaired subgroup (5 days) was longer than guideline recommendations [12, 42]. Practically, cognitively impaired patients required longer to recruit due to the requirement for a consultee declaration for consent from a family member or caregiver. Finally, we did not adjust for multiplicity in our analysis, so interpretation of secondary outcomes should be exploratory.

Among older patients with NSTEMI and cognitive impairment, an invasive treatment strategy did not reduce the cumulative incidence of cardiovascular death or nonfatal MI compared to a conservative treatment strategy. However, an invasive treatment strategy may reduce the cumulative incidence of nonfatal MI among older patients with NSTEMI and evidence of mild cognitive impairment at baseline compared to a conservative treatment strategy, although this finding should be interpreted as hypothesis-generating. There were no differences in primary and secondary outcomes among older patients with NSTEMI and normal cognition.

## Supplementary Material

Supplementary_materials_afag330
