# Supplementary material for: Invasive versus conservative strategy for older patients with myocardial infarction based on cognitive function: the SENIOR-RITA cognition analysis
Source: Age Ageing. 2026 Nov 12;55(11):afag330. doi: 10.1093/ageing/afag330 (PMC13755347; doi:10.1093/ageing/afag330)
Supplement: Supplementary file 1 [file supplementary_materials_afag330.docx]

**Invasive versus conservative strategy for older patients with myocardial infarction based on cognitive function: The SENIOR-RITA Cognition Analysis**

**Supplemental Material**

**TABLE OF CONTENTS**

[SUPPLEMENTAL METHODS	3](#_Toc236752471)

[Inclusion and exclusion criteria of the SENIOR-RITA Trial.	3](#_Toc236752472)

[Fried Frailty Index	4](#_Toc236752473)

[Charlson Comorbidity Index	6](#_Toc236752474)

[Montreal Cognitive Assessment (MoCA)	7](#_Toc236752475)

[SUPPLEMENTAL FIGURES	8](#_Toc236752476)

[Figure S1: Sub-study flow chart	8](#_Toc236752477)

[Figure S2: Distribution of MoCA scores	9](#_Toc236752478)

[Figure S3: Cumulative incidence of the primary outcome in normal cognition patients.	10](#_Toc236752479)

[Figure S4: Cumulative incidence of cardiovascular death in normal cognition patients.	11](#_Toc236752480)

[Figure S5: Cumulative incidence of non-fatal myocardial infarction in normal cognition patients.	12](#_Toc236752482)

[Figure S6. Interaction test between treatment strategy and cognitive function.	13](#_Toc236752483)

[SUPPLEMENTAL TABLES	14](#_Toc236752484)

[Table S1. Baseline demographics and clinical characteristics stratified by severity of cognitive impairment	14](#_Toc236752485)

[Table S2. Medical therapy and procedural characteristics in cognitively impaired patients as per randomised strategy	16](#_Toc236752486)

[Table S3. Baseline demographics and clinical characteristics of normal cognition patients as per randomised strategy	18](#_Toc236752487)

[Table S4. Medical therapy and procedural characteristics in normal cognition patients as per randomised strategy	20](#_Toc236752488)

[Table S5. Primary and secondary outcomes stratified by severity of cognitive impairment	22](#_Toc236752489)

[Table S6. Restricted mean event-free time to account for non-proportional hazards in cognitively impaired patients at 5-year follow-up	23](#_Toc236752490)

[Table S7. Restricted mean event-free time to account for non-proportional hazards in normal cognition patients at 5-year follow-up.	23](#_Toc236752491)

[Table S8. Sensitivity analyses for primary outcome	24](#_Toc236752492)

[Table S9: Procedural-related complications in cognitively impaired versus normal cognition patients	25](#_Toc236752493)

# **SUPPLEMENTAL METHODS**

Inclusion and exclusion criteria of the SENIOR-RITA Trial.

**Inclusion criteria (all the following)**

- Age ≥75 years old
- Type 1 NSTEMI during index hospitalization

**Exclusion criteria (any of the following)**

- Patients presenting with STEMI or unstable angina

- Patients with cardiogenic shock

- Patients with a known life expectancy of <1 year

- Patients in whom neither the patient nor the consultee are able and willing to provide written informed consent

- Previous randomization in the SENIOR RITA trial

- Inability to undergo invasive coronary angiography, such as no vascular access site, or absolute contraindication to coronary revascularization

Fried Frailty Index

| **Criterion Frailty Status** | **Criterion Frailty Status** |
| --- | --- |
| **Shrinking** | **Frailty cut point: Baseline:** Self-reported unintentional weight loss ≥10lb in previous year. **Follow-up:** Unintentional weight loss ≥5% of previous year’s body weight OR BMI <18.5kg/m^2^. |
| **Physical endurance/energy** | **Geriatric Depression Scale:** Do you feel full of energy? During the last 4 weeks how often you rested in bed during day? *Response options: Every day, every week, once, not at all.*  **Frailty cut point:** No to 1 and every day or every week to 2. |
| **Low physical activity** | Frequency of mildly energetic, moderately energetic and very energetic physical activity. *Response options: ≥3 times per week, 1-2 times per week, 1-3 times per month, hardly ever/never.*  **Frailty cut point:** Hardly ever/never for very energetic physical activity AND for moderately energetic physical activity. |
| **Weakness** | Hand grip strength in Kg: GRIP-D handheld dynamometer, dominant hand, average of 3 measures.  **Frailty cut point: Grip strength:** lowest 20% (by gender, body mass index) Men BMI ≤24 ≤29 BMI 24.1–26 ≤30 BMI 26.1–28 ≤30 BMI >28 ≤32 Women BMI ≤23 ≤17 BMI 23.1–26 ≤17.3 BMI 26.1–29 ≤18 BMI >29 ≤21 |
| **Slow walking speed** | Walking time in seconds (usual pace) over 15 feet.  **Frailty cut point:** Slowest 20%, stratified by gender and median standing height. Men Height ≤173 cm Height >173 cm Women Height ≤159 cm Height >159 cm ≥7 seconds ≥6 seconds ≥7 seconds ≥6 seconds. OR Time to complete “timed up and go test” (TUG).  **Frailty cut point:** TUG time ≥19 seconds. |
| **Frail:** ≥3 criteria present. **Intermediate or Pre-Frail:**1 or 2 criteria present. **Robust:** 0 criteria present. | |

Charlson Comorbidity Index

| **Each condition is assigned a score of 1, 2, 3, or 6.** | |
| --- | --- |
| **1** | Myocardial infarction, congestive heart failure, peripheral vascular disease, cerebrovascular disease, dementia, chronic pulmonary disease, rheumatologic disease, peptic ulcer disease, liver disease (if mild, or 3 if moderate/severe), diabetes (if controlled, or 2 if uncontrolled) |
| **2** | Hemiplegia or paraplegia, renal disease, malignancy (if localized, or 6 if metastatic tumor), leukemia, lymphoma |
| **6** | AIDS |
| **Age:** | |
| **+1** | 50-59 years old |
| **+2** | 60-69 years old |
| **+3** | 70-79 years old |
| **+4** | 80 years old or more |

Montreal Cognitive Assessment (MoCA)

| **Domain** | **Maximum Score** |
| --- | --- |
| Executive/visuospatial function | 5 points |
| Naming | 3 points |
| Attention | 6 points |
| Language | 3 points |
| Abstraction | 2 points |
| Recall | 5 points |
| Orientation | 6 points |
| TOTAL | 30 points |
| **1 point** is added to the total score if a person has 12 years or less of formal education | |
| Scores ≥26 is classified as normal, and <26 as cognitively impaired | |

# **SUPPLEMENTAL FIGURES**

Figure S1: Sub-study flow chart

MoCA denotes Montreal Cognitive Assessment

Figure S2: Distribution of MoCA scores

MoCA denotes Montreal Cognitive Assessment

Figure S3: Cumulative incidence of the primary outcome in normal cognition patients.

CI denotes confidence interval

Figure S4: Cumulative incidence of cardiovascular death in normal cognition patients.

CI denotes confidence interval

Figure S5: Cumulative incidence of non-fatal myocardial infarction in normal cognition patients.

CI denotes confidence interval

Figure S6. Interaction test between treatment strategy and cognitive function.

Hazard ratio is plotted on a ${log}_{10}$ scale.

P values are derived from Wald test for interaction.

# **SUPPLEMENTAL TABLES**

Table S1. Baseline demographics and clinical characteristics stratified by severity of cognitive impairment

| **Characteristics** | **Mild Cognitive Impairment**  **N = 754** | **Moderate Cognitive Impairment**  **N = 132** | **Severe Cognitive Impairment**  **N = 23** |
| --- | --- | --- | --- |
| Female sex - no. (%) | 336 (45) | 77 (58) | 13 (57) |
| Median Age – yr (IQR) | 82 (79-86) | 83.5 (80-87) | 84 (78-89) |
| ≥75 to <80 - no. (%) | 208 (28) | 28 (21) | 6 (26) |
| ≥80 to < 85 - no. (%) | 294 (39) | 50 (38) | 8 (35) |
| ≥85 to < 90 - no. (%) | 197 (26) | 35 (27) | 4 (17) |
| ≥90 to < 95 - no. (%) | 52 (7) | 17 (13) | 3 (13) |
| ≥95 - no. (%) | 3 (0.4) | 2 (1.5) | 2 (9) |
| Median days from admission to randomisation (IQR) | 2 (1-3) | 2 (1-4) | 2 (1-4) |
| Median Rockwood Frailty score (IQR) | 3 (2-4) | 4 (3-5) | 5 (4-6) |
| Frail (Rockwood score ≥ 5) - no. /total no. (%) | 166/754 (22) | 59/132 (45) | 14/23 (61) |
| Very fit (1) - no. /total no. (%) | 75/754 (9.9) | 9/132 (6.8) | 0/23 (0) |
| Well, without active disease (2) - no. /total no. (%) | 134/754 (18) | 14/132 (11) | 1/23 (4) |
| Well, with treated co-morbidities (3) - no. /total no. (%) | 209/754 (28) | 25/132 (19) | 2/23 (9) |
| Apparently vulnerable (4) - no. /total no. (%) | 170/754 (23) | 25/132 (19) | 6/23 (26) |
| Mildly frail (5) - no. /total no. (%) | 115/754 (15) | 32/132 (24) | 8/23 (35) |
| Moderately frail (6) - no. /total no. (%) | 45/754 (6.0) | 24/132 (18) | 5/23 (22) |
| Severely frail (7) - no. /total no. (%) | 6/754 (0.8) | 3/132 (2.3) | 1/23 (4) |
| Median Fried Frailty score (IQR) | 2 (1-3) | 2 (2-4) | 3 (2-4) |
| Robust (0) - no. /total no. (%) | 135/726 (19) | 9/127 (7) | 1/22 (5) |
| Pre-frail (1 or 2) - no. /total no. (%) | 339/726 (47) | 58/127 (46) | 8/22 (36) |
| Fried Frailty score Frail (≥3) - no. /total no. (%) | 252/726 (35) | 60/127 (47) | 13/22 (59) |
| Median Charlson age-adjusted co-morbidity index (IQR) | 5 (4-6) | 5 (5-7) | 5 (4-7) |
| Smoking status |  |  |  |
| Current Smoker - no. /total no. (%) | 31/749 (4) | 10/129 (8) | 4/23 (17) |
| Ex-Smoker - no. /total no. (%) | 341/749 (46) | 52/129 (40) | 10/23 (43) |
| Never Smoked - no. /total no. (%) | 377/749 (50) | 67/129 (52) | 9/23 (39) |
| Median GRACE 2 Score (IQR) | 137 (124-150) | 135 (124-156) | 145 (134-150) |
| Hypertension - no. /total no. (%) | 499/754 (66) | 93/132 (70) | 14/23 (61) |
| Diabetes - no. /total no. (%) | 210/754 (28) | 55/132 (42) | 4/23 (17) |
| Hypercholesterolemia - no. /total no. (%) | 232/754 (31) | 38/131 (29) | 6/23 (26) |
| History of chronic kidney disease - no. /total no. (%) | 156/754 (21) | 37/132 (28) | 4/23 (17) |
| Previous myocardial infarction - no. /total no. (%) | 265/754 (35) | 38/132 (29) | 9/23 (39) |
| Previous PCI - no. /total no. (%) | 173/754 (23) | 20/132 (15) | 6/23 (26) |
| Previous CABG - no. /total no. (%) | 94/754 (12) | 13/132 (10) | 3/23 (13) |
| History of peripheral vascular disease - no. /total no. (%) | 54/754 (7) | 14/132 (11) | 2/23 (9) |
| History of TIA/Stroke - no. /total no. (%) | 125/754 (17) | 26/132 (20) | 4/23 (17) |
| History of COPD - no. /total no. (%) | 122/754 (16) | 23/132 (17) | 4/23 (17) |
| History of Congestive Heart Failure - no. /total no. (%) | 83/754 (11) | 18/132 (14) | 3/23 (13) |
| History of Dementia – no. /total no. (%) | 10/754 (1) | 12/132 (9) | 6/23 (26) |

IQR denotes interquartile range, MoCA denotes Montreal Cognitive Assessment, PCI denotes percutaneous coronary intervention, CABG denotes coronary artery bypass graft, TIA denotes transient ischaemic attack and COPD denotes chronic obstructive pulmonary disease

Table S2. Medical therapy and procedural characteristics in cognitively impaired patients as per randomised strategy

| **DISCHARGE MEDICAL THERAPY** | **Invasive Strategy**  **(N=433)** | **Conservative Strategy (N=476)** | **P value** |
| --- | --- | --- | --- |
| Antiplatelet therapies |  |  |  |
| Aspirin - no. /total no. (%) | 387/432 (90) | 403/475 (85) | 0.033 |
| P2Y_12_ Receptor Antagonist (Total) - no. /total no. (%) | 389/432 (90) | 446/475 (94) | 0.032 |
| Clopidogrel - no. /total no. (%) | 214/432 (50) | 272/475 (57) | 0.020 |
| Ticagrelor - no. /total no. (%) | 173/432 (40) | 173/475 (36) | 0.30 |
| Prasugrel - no. /total no. (%) | 2/432 (0.5) | 1/475 (0.2) | 0.60 |
| Antiplatelet therapy |  |  | 0.5 |
| None - no. /total no. (%) | 0/432 (0) | 0/475 (0) |  |
| Single - no. /total no. (%) | 78/432 (18) | 95/475 (20) |  |
| Dual - no. /total no. (%) | 354/432 (82) | 380/475 (80) |  |
| Anticoagulant (Total) - no. /total no. (%) | 97/432 (22) | 121/475 (25) | 0.3 |
| Apixaban – no. /total no. (%) | 32/432 (7.4) | 49/475 (10) |  |
| Rivaroxaban - no. /total no. (%) | 28/432 (6.5) | 23/475 (4.8) |  |
| Warfarin - no. /total no. (%) | 13/432 (3.0) | 22/475 (4.6) |  |
| Edoxaban - no. /total no. (%) | 11/432 (2.5) | 10/475 (2.1) |  |
| Dabigatran - no. /total no. (%) | 0/432 (0) | 4/475 (0.8) |  |
| Other – no. /total no. (%) | 13/432 (3.0) | 13/475 (2.7) |  |
| Triple therapy (Total) - no. /total no. (%) | 56/432 (13) | 53/475 (11) | 0.4 |
| Lipid-lowering therapy - no. /total no. (%) | 394/432 (91) | 424/475 (89) | 0.7 |
| Atorvastatin - no. /total no. (%) | 343/432 (79) | 375/475 (79) |  |
| Simvastatin - no. /total no. (%) | 25/432 (5.8) | 26/475 (5.5) |  |
| Rosuvastatin - no. /total no. (%) | 16/432 (3.7) | 17/475 (3.6) |  |
| Pravastatin – no. /total no. (%) | 7/432 (1.6) | 3/475 (0.6) |  |
| Ezetimibe - no. /total no. (%) | 3/432 (0.7) | 3/475 (0.6) |  |
| ACE Inhibitor or ARB (Total) – no. / total no. (%) | 304/431 (71) | 313/474 (66) | 0.3 |
| Ramipril - no. /total no. (%) | 205/431 (48) | 210/474 (44) |  |
| Lisinopril - no. /total no. (%) | 35/431 (8.1) | 33/474 (7.0) |  |
| Perindopril - no. /total no. (%) | 13/431 (3.0) | 17/474 (3.6) |  |
| Candesartan - no. /total no. (%) | 24/431 (5.6) | 17/474 (3.6) |  |
| Other - no. /total no. (%) | 27/431 (6.3) | 36/474 (7.6) |  |
| Beta blocker (Total) – no. / total no. (%) | 338/431 (78) | 381/474 (80) | 0.5 |
| Bisoprolol - no. /total no. (%) | 323/431 (75) | 356/474 (75) |  |
| Atenolol - no. /total no. (%) | 8/431 (1.9) | 14/474 (3.0) |  |
| Other - no. /total no. (%) | 7/431 (1.6) | 11/474 (2.3) |  |
| **PROCEDURAL CHARACTERISTICS** |  |  |  |
| Angiography performed – no. (%) | 381 (88) | NA |  |
| Radial access – no. (%) | 340 (89.2) | NA |  |
| Multivessel disease – no./ total no. (%) | 212/378 (56) | NA |  |
| Median days from admission to angiography (IQR) | 5 (3-8) | NA |  |
| Median days from randomisation to angiography (IQR) | 3 (1-5) | NA |  |
| Reason not performed |  |  |  |
| Clinical decision – no. (%) | 24 (46.2) | NA |  |
| Participant decision – no. (%) | 16 (30.8) | NA |  |
| Participant too unwell – no. (%) | 10 (19.2) | NA |  |
| Participant died – no. (%) | 2 (3.8) | NA |  |
| Not known – no. (%) | 0 (0) | NA |  |
| Revascularisation performed – no. (%) |  |  |  |
| PCI – no. (%) | 190 (95) | NA |  |
| CABG – no. (%) | 11 (5) | NA |  |
| Median days from admission to PCI (IQR) | 5 (3-7) | NA |  |
| Median days from randomisation to PCI (IQR) | 2 (1-4) | NA |  |
| Median days from admission to CABG (IQR) | 19 (14-27) | NA |  |

ACE denotes angiotensin converting enzyme, ARB denotes Angiotensin receptor blocker, CABG denoted coronary artery bypass surgery, IQR denotes interquartile range and PCI denotes percutaneous coronary intervention.

Table S3. Baseline demographics and clinical characteristics of normal cognition patients as per randomised strategy

| **Characteristics** | **Invasive strategy**  **(N =291)** | **Conservative strategy**  **(N =255)** | **P value** |
| --- | --- | --- | --- |
| Female sex - no. (%) | 121 (42) | 106 (42) | >0.9 |
| Median age - yr (IQR) | 81 (78-84) | 81 (78-84) | 0.8 |
| ≥75 to <80 - no. (%) | 103 (35) | 93 (36) | >0.9 |
| ≥80 to < 85 - no. (%) | 116 (40) | 101 (40) |  |
| ≥85 to < 90 - no. (%) | 60 (21) | 47 (18) |  |
| ≥90 to < 95 - no. (%) | 9 (3.1) | 11 (4.3) |  |
| ≥95 - no. (%) | 3 (1.0) | 3 (1.2) |  |
| Median days from admission to randomisation (IQR) | 2 (1-3) | 2 (1-2) | 0.2 |
| Median Rockwood Frailty score (IQR) | 3 (2-4) | 3 (2-3) | 0.4 |
| Frail (Rockwood score ≥ 5) - no. /total no. (%) | 30/291 (10) | 25/255 (10) |  |
| Very fit (1) – no. /total no. (%) | 61/291 (21) | 52/255 (20) |  |
| Well, without active disease (2) - no. /total no. (%) | 61/291 (21) | 65/255 (25) |  |
| Well, with treated co-morbidities (3) - no. /total no. (%) | 88/291 (30) | 78/255 (31) |  |
| Apparently vulnerable (4) - no. /total no. (%) | 51/291 (18) | 35/255 (14) |  |
| Mildly frail (5) - no. /total no. (%) | 20/291 (6.9) | 20/255 (7.8) |  |
| Moderately frail (6) - no. /total no. (%) | 7/291 (2.4) | 5/255 (2.0) |  |
| Severely frail (7) - no. /total no. (%) | 3/291 (1.0) | 0/255 (0) |  |
| Median Fried Frailty score (IQR) | 1 (0-2) | 1 (0-2) | >0.9 |
| Robust (0) - no. /total no. (%) | 80/285 (28) | 75/248 (30) |  |
| Pre-frail (1 or 2) - no. /total no. (%) | 140/285 (49) | 116/248 (47) |  |
| Fried Frailty score Frail (≥3) - no. /total no. (%) | 65/285 (23) | 57/248 (23) |  |
| Median Charlson age-adjusted co-morbidity index (IQR) | 5 (4-6) | 5 (4-6) | 0.4 |
| Smoking status |  |  | 0.9 |
| Current Smoker - no. /total no. (%) | 17/289 (5.9) | 13/253 (5.1) |  |
| Ex-Smoker - no. /total no. (%) | 137/289 (47) | 125/253 (49) |  |
| Never Smoked - no. /total no. (%) | 135/289 (47) | 115/253 (45) |  |
| Median GRACE 2 Score (IQR) | 133 (120-147) | 135 (121-149) |  |
| Hypertension - no. /total no. (%) | 183/291 (63) | 164/255 (64) | 0.7 |
| Diabetes - no. /total no. (%) | 96/291 (33) | 77/255 (30) | 0.5 |
| Hypercholesterolemia - no. /total no. (%) | 93/290 (32) | 90/255 (35) | 0.4 |
| History of chronic kidney disease - no. /total no. (%) | 51/291 (18) | 46/255 (18) | 0.9 |
| Previous myocardial infarction - no. /total no. (%) | 76/291 (26) | 68/255 (27) | 0.9 |
| Previous PCI - no. /total no. (%) | 50/290 (17) | 40/255 (16) | 0.6 |
| Previous CABG - no. /total no. (%) | 33/291 (11) | 30/255 (12) | 0.9 |
| History of peripheral vascular disease - no. /total no. (%) | 18/291 (6.2) | 20/255 (7.8) | 0.4 |
| History of TIA/Stroke - no. /total no. (%) | 37/291 (13) | 28/255 (11) | 0.5 |
| History of COPD - no. /total no. (%) | 36/291 (12) | 33/255 (13) | 0.8 |
| History of Congestive Heart Failure - no. /total no. (%) | 16/291 (5.5) | 16/255 (6.3) | 0.7 |
| History of Dementia - no. /total no. (%) | 0/291 (0) | 1/255 (0.4) | 0.5 |

CABG denotes coronary artery bypass graft, COPD denotes chronic obstructive pulmonary disease, IQR denotes interquartile range, MoCA denotes Montreal Cognitive Assessment, PCI denotes percutaneous coronary intervention and TIA denotes transient ischaemic attack.

Table S4. Medical therapy and procedural characteristics in normal cognition patients as per randomised strategy

| **DISCHARGE MEDICAL THERAPY** | **Invasive strategy**  **(N = 291)** | **Conservative strategy**  **(N =255)** | **P value** |
| --- | --- | --- | --- |
| Antiplatelet therapies |  |  |  |
| Aspirin - no. /total no. (%) | 267/291 (92) | 235/255 (92) | 0.9 |
| P2Y_12_ Receptor Antagonist (Total) - no. / total no. (%) | 261/291 (90) | 243/255 (95) | **0.014** |
| Clopidogrel - no. /total no. (%) | 116/291 (40) | 110/255 (43) | 0.4 |
| Ticagrelor - no. /total no. (%) | 144/291 (49) | 133/255 (52) | 0.5 |
| Prasugrel - no. / total no. (%) | 1/291 (0.3) | 0/255 (0) | >0.9 |
| Antiplatelet therapy |  |  | 0.3 |
| None - no. /total no. (%) | 0/291 (0) | 0/255 (0) |  |
| Single - no. /total no. (%) | 44/291 (15) | 30/255 (12) |  |
| Dual - no. /total no. (%) | 247/291 (85) | 225/255 (88) |  |
| Anticoagulant (Total) - no. (%) | 67/291 (23) | 51/255 (20) | 0.4 |
| Apixaban – no. /total no. (%) | 16/291 (5.5) | 16/255 (6.3) |  |
| Rivaroxaban - no. /total no. (%) | 14/291 (4.8) | 11/255 (4.3) |  |
| Warfarin - no. /total no. (%) | 15/291 (5.2) | 11/255 (4.3) |  |
| Edoxaban - no. /total no. (%) | 5/291 (1.7) | 5/255 (2.0) |  |
| Dabigatran - no. /total no. (%) | 1/291 (0.3) | 0/255 (0) |  |
| Other – no. /total no. (%) | 16/291 (5.5) | 8/255 (3.1) |  |
| Triple therapy (Total) - no. / total no. (%) | 38/291 (13) | 33/255 (13) | >0.9 |
| Lipid-lowering therapy -no. /total no. (%) | 265/291 (91) | 233/291 (91) | 0.9 |
| Atorvastatin - no. /total no. (%) | 233/291 (80) | 204/255 (80) |  |
| Simvastatin - no. /total no. (%) | 13/291 (4.5) | 16/255 (6.3) |  |
| Rosuvastatin - no. /total no. (%) | 13/291 (4.5) | 7/255 (2.7) |  |
| Pravastatin – no. /total no. (%) | 5/291 (1.7) | 6/255 (2.4) |  |
| Ezetimibe - no. /total no. (%) | 1/291 (0.3) | 0/255 (0) |  |
| ACE Inhibitor or ARB (Total) – no. / total no. (%) | 213/291 (73) | 176/255 (69) | 0.3 |
| Ramipril - no. /total no. (%) | 154/291 (53) | 120/255 (47) |  |
| Lisinopril - no. /total no. (%) | 22/291 (7.6) | 20/255 (7.8) |  |
| Perindopril - no. /total no. (%) | 11/291 (3.8) | 10/255 (3.9) |  |
| Candesartan - no. /total no. (%) | 12/291 (4.1) | 9/255 (3.5) |  |
| Other - no. /total no. (%) | 14/291 (4.8) | 17/255 (6.7) |  |
| Beta blocker (Total) – no. / total no. (%) | 235/291 (81) | 195/254 (77) | 0.2 |
| Bisoprolol - no. /total no. (%) | 214/291 (74) | 178/254 (70) |  |
| Atenolol - no. /total no. (%) | 11/291 (3.8) | 12/254 (4.7) |  |
| Other - no. /total no. (%) | 10/291 (3.4) | 5/254 (2.0) |  |
| **PROCEDURAL CHARACTERISTICS** |  | - |  |
| Angiography performed – no. (%) | 275 (91) | - |  |
| Radial access – no. (%) | 246 (89.5) | - |  |
| Multivessel disease – no./ total no. (%) | 158/274 (58) | - |  |
| Median days from admission to angiography (IQR) | 5 (3-7) | - |  |
| Median days from randomisation to angiography (IQR) | 3 (1-4) | - |  |
| Reason not performed |  | - |  |
| Clinical decision – no. (%) | 9 (56.3) | - |  |
| Participant decision – no. (%) | 4 (25) | - |  |
| Participant too unwell – no. (%) | 2 (12.5) | - |  |
| Participant died – no. (%) | 0 (0) | - |  |
| Not known – no. (%) | 1 (6.3) | - |  |
| Revascularisation performed – no. (%) | 163 (56) | - |  |
| PCI – no. (%) | 150 (92) | - |  |
| CABG – no. (%) | 13 (8) | - |  |
| Median days from admission to PCI (IQR) | 5 (3,7) | - |  |
| Median days from randomisation to PCI (IQR) | 3 (1,4) | - |  |
| Median days from admission to CABG (IQR) | 14 (13,29) | - |  |

Table S5. Primary and secondary outcomes stratified by severity of cognitive impairment

| **Outcome variables** | **Mild cognitive impairment**  **N = 754** | **Moderate or severe cognitive impairment**  **N = 155** |
| --- | --- | --- |
| Primary outcome and its components: Cardiovascular death or non-fatal MI - Hazard Ratio, 95% CI | 0.88 (0.66 to 1.16) | 0.71 (0.41 to 1.24) |
| Cardiovascular death - Hazard Ratio, 95% CI | 1.09 (0.76 to 1.55) | 0.74 (0.35 to 1.56) |
| Non-fatal MI - Hazard Ratio, 95% CI | 0.62 (0.41 to 0.94) | 0.61 (0.26 to 1.44) |
| Secondary outcomes |  |  |
| All cause death or non-fatal MI – Hazard Ratio, 95% CI | 0.93 (0.74 to 1.15) | 0.76 (0.49 to 1.19) |
| All cause death – Hazard Ratio, 95% CI | 1.08 (0.85 to 1.37) | 0.85 (0.52 to 1.37) |
| Non-cardiovascular death – Hazard Ratio, 95% CI | 1.13 (0.82 to 1.57) | 0.95 (0.53 to 1.70) |
| Fatal or non-fatal MI – Hazard Ratio, 95% CI | 0.64 (0.43 to 0.94) | 0.74 (0.33 to 1.66) |
| Coronary angiography – Hazard Ratio, 95% CI | 0.18 (0.11 to 0.30) | 0.15 (0.03 to 0.69) |
| Coronary revascularisation – Hazard Ratio, 95% CI | 0.23 (0.12 to 0.44) | 0.12 (0.02 to 0.98) |
| Stroke – Hazard Ratio, 95% CI | 0.64 (0.32 to 1.27) | 0.68 (0.20 to 2.33) |
| Transient ischaemic attack – Hazard Ratio, 95% CI | 1.44 (0.50 to 4.16) | 2.47 (0.22 to 27.3) |
| Hospitalisation for heart failure – Hazard Ratio, 95% CI | 1.22 (0.79 to 1.90) | 0.60 (0.23 to 1.55) |
| Bleeding (BARC Type 2+) – Hazard Ratio, 95% CI | 1.20 (0.70 to 2.06) | 1.85 (0.50-6.92) |

Hazard ratios are derived from Cox proportional hazards models, adjusted for Rockwood Clinical Frailty Scale and Charlson Comorbidity Index at randomisation.

Mild cognitive impairment is classed as MoCA 18-25

Moderate cognitive impairment is classed as MoCA 10-17

Severe cognitive impairment is classed as MoCA <10

Due to small numbers of patients with severe cognitive impairment, this has been combined with moderate cognitive impairment.

Table S6. Restricted mean event-free time to account for non-proportional hazards in cognitively impaired patients at 5-year follow-up

| **Outcome variables** | **Invasive Strategy^*^**  **(N=433)** | **Conservative Strategy^*^**  **(N=476)** | **Mean difference^†^ (95% CI)** |
| --- | --- | --- | --- |
| Primary Outcome | 3.85 (3.67-4.03) | 3.66 (3.48-3.85) | +68 days  (-27 days to +163 days) |
| Cardiovascular death | 4.22 (4.07-4.38) | 4.24 (4.08-4.39) | -5 days  (-85 days to +75 days) |
| All-cause death or non-fatal MI | 3.28 (3.10-3.47) | 3.14 (2.95-3.33) | +52 days  (-45 days to +148 days) |
| Time-to-first non-trial coronary angiography | 4.79 (4.69-4.88) | 3.91 (3.72-4.09) | +322 days  (+246 days to +398 days) |

^*^Data shown are the restricted mean event-free time at 5 years post-randomisation (95% confidence intervals). ^†^Mean difference for invasive strategy compared to conservative strategy, estimated in days. The widths of the confidence intervals have not been adjusted for multiplicity and should not be used in place of hypothesis testing.

Table S7. Restricted mean event-free time to account for non-proportional hazards in normal cognition patients at 5-year follow-up.

| **Outcome variables** | **Invasive Strategy^*^**  **(N=291)** | **Conservative Strategy^*^**  **(N=255)** | **Mean difference^†^ (95% CI)** |
| --- | --- | --- | --- |
| Primary Outcome | 4.08 (3.88-4.28) | 4.25 (4.05-4.45) | -62 days  (-167 days to +42 days) |
| Cardiovascular death | 4.56 (4.41-4.71) | 4.72 (4.60-4.85) | -60 days  (-132 days to +11 days) |
| All-cause death or non-fatal MI | 3.64 (3.42-3.85) | 3.81 (3.59-4.04) | -65 days  (-180 days to +50 days) |
| Time-to-first non-trial coronary angiography | 4.69 (4.56-4.82) | 3.63 (3.36-3.90) | +388 days  (+279 days to +497 days) |

^*^Data shown are the restricted mean event-free time at 5 years post-randomisation (95% confidence intervals). ^†^Mean difference for invasive strategy compared to conservative strategy, estimated in days.
The widths of the confidence intervals have not been adjusted for multiplicity and should not be used in place of hypothesis testing.

Table S8. Sensitivity analyses for primary outcome

| **Sensitivity analysis*** | **Cognitive impairment^†^** | | **Normal cognition^†^** | |
| --- | --- | --- | --- | --- |
| a) Competing risk analysis | Invasive Strategy  N = 433 | Conservative Strategy  N = 476 | Invasive Strategy  N = 291 | Conservative Strategy  N = 255 |
|  | 0.85 (0.67-1.09) | | 1.20 (0.83-1.75) | |
| b) Multiple imputation chained equations to account for missing MoCA data | Invasive Strategy  N = 456 | Conservative Strategy  N = 502 | Invasive Strategy  N = 296 | Conservative Strategy  N = 262 |
|  | 0.88 (0.69-1.12) | | 1.15 (0.80-1.66) | |

^*^All sensitivity analyses are conducted with respect to the primary outcome of cardiovascular death or non-fatal myocardial infarction

a) Fine and Gray sub-distribution hazard ratio according to Aalen-Johansen estimates, whereby non-cardiovascular death is modelled as a competing risk

b) Multiple imputation chained equations to account for missing MoCA data. We utilised 5 imputations, imputed missing MoCA data (<5%) using predictive mean matching, and accounted for the interactions of randomised treatment strategy and Fried Frailty Index.

^†^ Results are presented as hazard ratio (95% confidence interval)

Table S9: Procedural-related complications in cognitively impaired versus normal cognition patients

| **Procedural Complication** | **Cognitive impairment**  **N = 433** | **Normal cognition**  **N = 291** | **P value** |
| --- | --- | --- | --- |
| Bleeding (BARC criteria) – no. (%) | 6 (1.4) | 4 (1.4) | >0.9 |
| Type 1 | 1 (0.2) | 1 (0.3) |  |
| Type 2 | 4 (0.9) | 3 (1.0) |  |
| Type 3a | 0 | 0 |  |
| Type 3b | 0 | 0 |  |
| Type 3c | 0 | 0 |  |
| Type 4 | 0 | 0 |  |
| Type 5a | 0 | 0 |  |
| Type 5b | 1 (0.2) | 0 |  |
| Coronary Dissection - no. (%) | 4 (0.9) | 1 (0.3) | 0.7 |
| Myocardial Infarction - no. (%) | 1 (0.2) | 3 (1.0) | 0.3 |
| >25% increase in serum creatinine concentration - no. (%) | 2 (0.5) | 1 (0.3) | >0.9 |
| Vascular complications needing intervention - no. (%) | 1 (0.2) | 2 (0.7) | 0.6 |
| Other – Cardiac arrest - no. (%) | 1 (0.2) | 2 (0.7) | 0.3 |
| Emergency repeat angiography or PCI - no. (%) | 2 (0.5) | 0 (0) |  |
| Other – Cardiogenic shock - no. (%) | 0 (0) | 1 (0.4) |  |
| Other – Vasovagal event - no. (%) | 0 (0) | 2 (0.8) |  |
| Other – Stable chest pain - no. (%) | 2 (0.5) | 0 (0) |  |
| TIA - no. (%) | 0 (0) | 1 (0.4) | 0.4 |
| Stroke - no. (%) | 1 (0.2) | 0 (0) | >0.9 |
| Death - no. (%) | 1 (0.2) | 0 (0) | >0.9 |
| Other – Contrast reaction - no. (%) | 0 | 0 | 0.3 |
| Other – Stent dislodgement - no. (%) | 1 (0.2) | 0 (0) |  |
| Other – Confusion and agitation - no. (%) | 1 (0.2) | 0 (0) |  |

BARC denotes Bleeding Academic Research Consortium, NA denotes not available, PCI denotes percutaneous coronary intervention, and TIA denotes transient ischaemic attack.
